# Enhanced Salt Tolerance Conferred by the Complete 2.3 kb cDNA of the Rice Vacuolar Na^+^/H^+^ Antiporter Gene Compared to 1.9 kb Coding Region with 5′ UTR in Transgenic Lines of Rice

**DOI:** 10.3389/fpls.2016.00014

**Published:** 2016-01-25

**Authors:** U. S. M. Amin, Sudip Biswas, Sabrina M. Elias, Samsad Razzaque, Taslima Haque, Richard Malo, Zeba I. Seraj

**Affiliations:** Plant Biotechnology Laboratory, Department of Biochemistry and Molecular Biology, University of DhakaDhaka, Bangladesh

**Keywords:** role of UTR, transgenic plants, salinity tolerant, *OsNHX1*, rice landraces

## Abstract

Soil salinity is one of the most challenging problems that restricts the normal growth and production of rice worldwide. It has therefore become very important to produce more saline tolerant rice varieties. This study shows constitutive over-expression of the vacuolar Na^+^/H^+^ antiporter gene (*OsNHX1*) from the rice landrace (Pokkali) and attainment of enhanced level of salinity tolerance in transgenic rice plants. It also shows that inclusion of the complete un-translated regions (UTRs) of the alternatively spliced *OsNHX1* gene provides a higher level of tolerance to the transgenic rice. Two separate transformation events of the *OsNHX1* gene, one with 1.9 kb region containing the 5′ UTR with CDS and the other of 2.3 kb, including 5′ UTR, CDS, and the 3′ UTR regions were performed. The transgenic plants with these two different constructs were advanced to the T_3_ generation and physiological and molecular screening of homozygous plants was conducted at seedling and reproductive stages under salinity (NaCl) stress. Both transgenic lines were observed to be tolerant compared to WT plants at both physiological stages. However, the transgenic lines containing the CDS with both the 5′ and 3′ UTR were significantly more tolerant compared to the transgenic lines containing *OsNHX1* gene without the 3′ UTR. At the seedling stage at 12 dS/m stress, the chlorophyll content was significantly higher (*P* < 0.05) and the electrolyte leakage significantly lower (*P* < 0.05) in the order 2.3 kb > 1.9 kb > and WT lines. Yield in g/plant in the best line from the 2.3 kb plants was significantly more (*P* < 0.01) compared, respectively, to the best 1.9 kb line and WT plants at stress of 6 dS/m. Transformation with the complete transcripts rather than the CDS may therefore provide more durable level of tolerance.

## Introduction

Salinity is one of the prime factors for deterioration of the agricultural crop production scenario. Soil salinity has adverse effects on plant germination, strength, and yield (Munns and Tester, 2008). The consequences of excessive salinity have already been reported for more than 45 million hectares of irrigated lands worldwide. Each year, about 1.5 million hectares of cultivable lands are becoming agriculturally unfavorable due to high salinity levels (Munns and Tester, 2008; Carillo et al., 2011). Elevated salinity affects plants in several ways with two main components, an initial osmotic stress and a subsequent accumulation of toxic ions (Munns et al., 2006). Long-term exposure to salinity leads to ionic stress, which can cause premature senescence of adult leaves and reduce the photosynthetic area available to support continued growth (Cramer and Nowak, 1992; Munns, 2002; Chinnusamy et al., 2005; Allu et al., 2014; Álvarez and Sánchez-Blanco, 2014). Plants need the ability to transport, compartmentalize, extrude, and mobilize Na^+^ ions to escape the detrimental effect of salinity stress (Apse and Blumwald, 2007). This compartmentalization also allows plants to use NaCl as an osmoticum to maintain osmotic potential that drives water into the cells (Blumwald et al., 2000).

Vacuolar Na^+^/H^+^ antiporters can sequester excess sodium ions from the cytosol into the vacuole when plants consume high levels of sodium salt (Fukuda et al., 1999). This Na^+^ transport is driven by the electrochemical gradient of proton generated by the vacuolar H^+^ translocating enzymes like H^+^/ATPase and H^+^-PPase. Vacuolar NHX transporters have shown to play significant roles in endosomal pH regulation (Yamaguchi et al., 2001), cellular K^+^ homeostasis and cell expansion (Apse et al., 2003), vesicular trafficking and protein targeting (Bowers et al., 2000; Sottosanto et al., 2004; Brett et al., 2005). NHX antiporters have also been reported as the main mediator of cytosolic K^+^ uptake into the vacuole and the switch between Na^+^ and K^+^ transport is regulated by calcium mediated calmodulin-like proteins responsive to the increasing pH under saline conditions (Yamaguchi et al., 2005).

Vacuolar NHX*s* have been shown to be important determinants of salt tolerance in plants (Rodríguez-Rosales et al., 2009; Schroeder et al., 2013). Genome sequencing projects have now shown that plants contain a very large number of putative vacuolar Cation/Proton antiporters. Eight NHX homologs have been identified in *Arabidopsis* so far and grouped on the basis of function and sequence similarity into three different classes like; vacuolar antiporters (NHX1, NHX2, NHX3, and NHX4), plasma membrane antiporters (NHX7/SOS1 and NHX8) and endosomal/vesicular antiporters (NHX5 and NHX6) (Bassil et al., 2012; Reguera et al., 2014). The nomenclature in rice under NHX, includes five different isoforms, all of which are vacuolar antiporters (subdivided in two groups based on sequence similarities like NHX1- NHX4 and NHX5) reported to date (Fukuda et al., 2011). In addition, the rice *OsNHX1* antiporter gene produces three different transcripts where transcript 1 and 2 produce proteins of equal size, while transcript 3 produces a truncated protein at the C terminus region. The sizes of these transcripts are 2265, 2394, and 1820 bp, respectively. However, the 5′ Un-Translated region (UTR) is variable in all three transcripts of the *OsNHX1* gene (196, 325, and 186 bp in transcript 1, 2, and 3, respectively) (LOC_Os07g47100, gramene). The 3′ UTR in Transcript 1 and 2 are the same 461 bp whereas in Transcript 3, it is only 311 bp. There is no reported alternative splicing in the *Arabidopsis thaliana* vacuolar antiporters.

The UTR of a gene has important biological roles which can influence the half-life, intracellular localization, and differential translational efficiency of the corresponding mRNA (Pesole et al., 2000; Mignone et al., 2002; Mazumder et al., 2003; Chabanon et al., 2004; de Moor et al., 2005). The properties of a transcript are controlled by some features of their UTRs. These features are the structures of stem-loop, initiation codons (upstream) and open reading frames (ORFs), various *cis*-acting elements and internal ribosome entry sites which are bound by RNA-binding proteins (Mignone et al., 2002). Moreover, differential translational regulation mediated by the UTRs is involved in metabolism, stress response, development, differentiation, and many other important biological processes (Brenneis and Soppa, 2009). The genomic regions corresponding to the UTRs can include introns, more commonly in the 5′ than in the 3′ UTR, which can often show differential splice pattern to produce alternative transcripts of different length. The 3′ UTRs contain both binding regions for regulatory proteins and microRNA target sites. The regulatory proteins binding regions at the 3′ UTR helps stabilize and localize the mRNA (Doroshenk et al., 2014). Different length of nucleotides at the 3′ UTR of *A. thaliana* dicer-like protein 2 (DCL2) was found to be associated with different levels of its transcripts (He et al., 2012). Alternative splicing events where two forms were equally prevalent, were found to be mainly controlled by untranslated regions (English et al., 2010). An important role of both 5′ and 3′ untranslated regions has been reported in the regulation of serine/arginine-rich (SR) protein gene expression. These proteins play important developmental roles during environmental stress (Rauch et al., 2014).

The vacuolar Na^+^/H^+^ antiporter gene has been transformed for salinity tolerance to many plants like *Arabidopsis* (Apse et al., 1999), rice (Biswas et al., 2015), tomato (Zhang and Blumwald, 2001), maize (Yin et al., 2004), wheat (Xue et al., 2004), and brassica (Rajagopal et al., 2007), etc. The quoted works reported high tolerance in dicots like, tomato, and brassica, where the plants set flowers and near normal seeds in ∼200 mM salt and the source of the genes were from *A. thaliana* (Zhang and Blumwald, 2001) and *Pennisetum glaucum* (Rajagopal et al., 2007). However in the case of monocots, tolerance was mostly reported only at the seedling level (Fukuda et al., 2004; Yin et al., 2004; Wu et al., 2005; Chen et al., 2007) or at a moderate level at the reproductive stage (Xue et al., 2004; Verma et al., 2007; Biswas et al., 2015). In the case of these cereals, the NHX1 gene was from *A. thaliana* (Xue et al., 2004; Yin et al., 2004), from *P. glaucum* (Verma et al., 2007) and from rice (Fukuda et al., 2004; Wu et al., 2005; Chen et al., 2007; Biswas et al., 2015). Previous workers overexpressing *OsNHX1* in rice have used only the coding region (CDS) for transformation and reported a range of slight to moderate improvement in seedling salt tolerance levels over WT, but no reproductive stage tolerance was discussed (Fukuda et al., 2004; Chen et al., 2007). Biswas et al. (2015) reported moderate level of tolerance of rice at the reproductive stage by using the CDS and the 5′ UTR of the Nipponbare rice Na^+^/H^+^ antiporter (1.9 kb). The present study reports the over-expression of vacuolar Na^+^/H^+^ antiporter from two events, one using the CDS and 5′ UTR (1.9 kb) and the other, the complete cDNA (2.3 kb) from a salt tolerant rice landrace *Pokkali* in a tissue culture responsive rice variety, Binnatoa (BA). The study includes generation of transgenic lines with two separate vectors constructed with transcript 2 of the *OsNHX1* gene; both were driven by the constitutive *CaMV35S* promoter. First vector was constructed with the 5′ UTR and CDS (1.9 kb) and the second with both the 5′ and 3′ UTRs and CDS (2.3 kb). The transgenic plants transformed with these two different constructs were advanced to the T_3_ generation and their molecular and physiological characterization done in both seedling and reproductive stages under salt stress. Moderate level of tolerance above WT was obtained in seedling and reproductive stages containing both constructs, but significant enhancement in salt tolerance was observed in the transgenic plants with the second construct containing both the UTRs and the CDS compared to the first construct without the 3′ UTR, thus indicating a role of 3′ UTR for better tolerance mediated by *OsNHX1* in transgenic rice.

## Materials and Methods

### Construction of *pENTR-DTOPO-CaMV35S-OsNHX1*

Total RNA was isolated from 16 days-old salt (NaCl) stressed (100 mM) *O. sativa* cv. Pokkali using Trizol and first strand cDNA was synthesized using oligodT following manufacturer’s protocol (Invitrogen, USA). PCR was performed to amplify the target sequences of *OsNHX1* transcript 2 with designed primers (**Supplementary Material 1.1**). Primer sets were designed for the whole fragments (2.3 kb) of *OsNHX1* (transcript 2) and complete CDS with 5′ UTR (1.9 kb) as mentioned by Fukuda et al. (1999). This comprised 294+1608+409 bp and 294+1608 bp, respectively (**Supplementary Material 1.2**). The forward primers for both cases were designed adding *CACC* overhang at the 5′ end to ensure compatibility while cloning into the entry vector (*pENTR-D-TOPO*). PCR reactions for both the target fragments were performed at 95°C for 5 min, 35 cycles of 1 min at 95°C, 30 s at 60°C, 2 min at 72°C followed by a final extension of 10 min at 72°C. The resulting fragments were gel purified using Qiagen Gel Purification system and later cloned to *pENTR-D-TOPO* vector following the manufacturer’s protocol (Invitrogen, USA). Positive clones were initially selected based on the lysate PCR confirmation from *kanamycin* resistant clones of transformed *E. coli* cells. Plasmid isolation was done from confirmed colonies using Promega plasmid isolation kit (Promega, USA). Isolated plasmids were digested with *Eco*R1 and *Eco*RV to check the insertion of the desired fragments into the entry vector. Then, digestion-positive colonies were sequenced with M13 forward and reverse primers by First Base DNA sequencing services from Malaysia. Clones with the confirmed sequences were then recombined with the destination vector (*pH7WG2.0*) by LR reaction (Invitrogen, USA) (Karimi et al., 2002). Positive clones were selected by gene specific PCR and restriction digestion (details not provided).

### Transformation of the Constructs into Rice Plants

*Agrobacterium tumefaciens* (LBA4404) was electroporated with the designed constructs *CaMV35S -OsNHX1 (1.9)* or *CaMV-OsNHX1(2.3)* in *pH7WG2.0* by applying standard protocols (Sambrook et al., 1989). The insertions were also confirmed by gene specific PCR and restriction digestion. *Agrobacterium* strains containing the desired constructs were then used to infect the transformation responsive rice calli of Bangladeshi rice Binnatoa (BA) following the protocol mentioned by Rasul et al. (1997), Khanna and Raina (1999). Transformed plants were regenerated to produce T_0_ plants. Positive T_0_ plants were advanced up to T_3_, based on germination in *Hygromycin* and PCR tests.

### Molecular and Physiological Screening of Transgenic Plants

#### PCR and RT-PCR

DNA was extracted from rice plant leaves using CTAB method (Doyle and Doyle, 1987). PCR was performed initially to detect the integration of the transgene based on the *hygromycin phosphotransferase* (hpt) gene present in the T-DNA region of the designed constructs in the transformed plants. PCR analyses were carried out in a 25 μl reaction mixture containing 100 ng of plant DNA, 100 μM of each dNTP, 2.4 ng each of primers: *HPT_F* and *HPT_R* (**Supplementary Material 1.1**), 1 unit of Taq DNA polymerase (Invitrogen, USA), 1.5 mM MgCl_2_, DMSO 2.4%, and 1 × PCR Buffer-MgCl_2_ (Invitrogen, USA). The optimized reaction was: Initial denaturation at 94°C (5 min), 35 cycles of denaturation at 94°C for 1 min, annealing at 61.2°C for 1 min and extension at 72°C for 1 min following a final extension at 72°C for 7 min. PCR was also done with gene specific primers (**Supplementary Material 1.1**) to confirm the integration of the desired fragments. Semi-quantitative RT-PCR was performed with RNA from both transgenic lines (containing *CaMV-OsNHX1-1.9* and *CaMV-OsNHX1-2.3*) at T_2_ stage following the protocol mentioned by Amin et al. (2012). *Glycerol 3-phosphate dehydrogenase* (*G3PDH*) and U6 snRNA were used here as control (house-keeping genes) for the semi-quantitative RT-PCR experiments (**Supplementary Material 1.1**). Semi-quantitative RT-PCR was also done in *CaMV-OsNHX1-1.9* and *CaMV-OsNHX1-2.3* plants as above using OsNHX1-transcript 3-specific primers in order to see whether there was any effect of the overexpression of transcript 2 on transcript 3 abundance (**Supplementary Material 1.1**).

#### *Hygromycin* Assay

Seeds from both transgenic lines at the T_0_ generation (with 1.9 and 2.3 kb fragments) and WT Binnatoa were incubated at 37°C O/N. The next day, seeds were incubated in *hygromycin* solution (50 mg/100 mL H_2_O) and kept at 37°C O/N followed by an incubation at room temperature for 6 days till the seeds germinated. Vigorously germinating seeds were initially considered as transformed lines and further tested.

#### Southern Blot Hybridization

Genomic DNA (20 μg) from the transgenic PCR positive T_3_ plants were digested with *Bam*H1. The enzyme was chosen strategically to get a band above 2 kb if there is an insertion of the transgene in the putative transformed plants, so the number of bands obtained should therefore depict the number of copies inserted in the genome. As positive control the whole plasmid *pH7WG2_NHX1_2.3* form the first construct was used after a single cut with *Bam*H1 and the plasmid was diluted to the amount of a single copy before loading into the gel. After electrophoresis, the DNAs were blotted onto a nylon membrane and probed using DIG-labeled PCR amplified product 809 bp with hpt gene- (CDS region) specific primers following standard protocol (Roche Diagnostics Inc., Mannheim, Germany).

#### Northern Blot Hybridization

Northern hybridization was carried out using two different probes. One was designed from the region specific for transcript 2 from the 5′ UTR and another from a common region spanning the coding sequence of all the transcripts (679 bp) (**Supplementary Material 1.1**). Transcript 2-specific northern was carried out on the transgenic plants, wild type (WT) Binnatoa and a highly salinity tolerant Indian rice landrace Pokkali, as well as from sensitive Nipponbare. RNA was isolated using Trizol and 20 μg of the RNA was used for loading. Nylon membranes and upward capillary transfer system was used for Northern using the DIG labeled probes according to the manufacturer’s protocol (Roche Diagnostics Inc., Mannheim, Germany).

#### Measurement of Chlorophyll Content and Leaf Senescence Assay

Fresh leaves were cut into pieces and 100 mg put into a bottle containing 12 ml of 80% acetone. After 48 h, absorbance of leaf tissue extract was measured at wavelength 663 and 645 nm. The total amount of chlorophyll content was calculated using formula: [{(0.00802 × A_663_) + (0.0202 × A_645_)}× V/W}]; A = absorbance, V = volume, and W = weight (Chutia and Borah, 2012). Leaves of non-transgenic and transgenic plants were cut into pieces for the leaf senescence assay and soaked in 100 and 200 mM NaCl solution and the senescence of the leaves were observed after 5 days.

#### Measurement of Electrolyte Leakage

Relative electrolyte leakage was measured according to (Cao et al., 2007) but refined according to (Parvin et al., 2015). The plant leaf segments were weighed (0.1 g) and taken in a falcon tube with 25 ml deionized water. The tubes were shaken on a gyratory shaker at room temperature for 2 h. The initial electrical conductivity (C1) of the solution was measured by using a conductivity detector. The leaf samples were then boiled in deionized water at 120°C for 10 min to release all the electrolytes from the tissues completely. The final electrical conductivity (C2) of the resulting solution was recorded. The percentage of electrolyte leakage was calculated according to the formula: (C1/C2) × 100. Finally statistical analysis was done by using t test.

#### Assessment of Salinity Tolerance at the Seedling Stage

The phenotypic screening for the salinity tolerance at seedling stage was done following the method described by Amin et al. (2012). Screening was done on the T_3_ population of both *CaMV-OsNHX1-1.9* and *CaMV-OsNHX1-2.3* plants. WT BA, salt tolerant control Pokkali, and salt sensitive IR29 were used for the screening. Sprouted seeds were sown in netted and floated styrofoam in PVC trays containing 10L Yoshida solution (Yoshida et al., 1976). The germinated seeds (9 for each line in 11 rows) were allowed to grow for 14 days. Then, NaCl stress was applied gradually starting from 4 dS/m to 12 dS/m at 24 h increments of 2 dS/m. An increase in leaf number and length was measured after an interval of 4 days for 16 days until 90% of the leaves of the sensitive control were damaged. The tolerance-related traits (LDS, root length, shoot length and leaf width) of all stressed plants were then measured. Data for percent survival and total leaf area affected was recorded according to the standard evaluation system of rice at IRRI (Gregorio et al., 1997). The level of salinity tolerance was evaluated mainly based on the value of LDS, which is based on the percentage of leaf damage. The plants were scored according to the protocol mentioned by Amin et al. (2012). Here the score of 1-9 corresponds from highly tolerant to extremely sensitive, respectively. The chlorophyll content and the dry weight of the stressed and control transgenic shoots as well as WT were measured at this stage (Vernon, 1960). For measuring dry weight, samples were kept at 70°C for 72 h in a hot-air circulating oven (Honeywell, UK model DT200).

#### Assessment of Salinity Tolerance at the Reproductive Stage

Reproductive stage screening was performed following the protocol in Amin et al. (2012). After 18 days in the hydroponic system in Yoshida solution, 30-day-old WT and transgenic lines were transferred individually to soil contained in perforated pots, where the soil was encased in a thin porous cloth to prevent leakage. The pots were placed in bowls of water, with five pots in each bowl. Each pot contained one plant. Each bowl therefore contained WT, sensitive control and the three transgenic plants. After 10 days, the pots were taken out of the water and allowed to drain for 24 h, and then transferred to large bowls filled with 6 dS/m NaCl solution in seven bowls or seven biological replicates. Water instead of NaCl solution was used in three more bowls to serve as control. The experiment was set up in a net house where the average temperature and humidity were 29°C and 72%, respectively. At maturity, plant height was measured with a meter scale from the base of each plant up to the tip of the panicle. At the end of reproductive stage, seeds were collected from transgenic and non-transgenic plants and the weight of total filled grains (g/plant) was measured to determine the grain yield. Other yield-related traits (tiller number, panicle number, panicle length, spikelet number, and spikelet fertility in percent) were also measured.

#### Measurement of Na^+^, K^+^ Content

Transgenic as well as the WT and other control plants were washed in flowing tap water for 30 s and oven-dried for the measurement of sodium and potassium concentrations in seedling shoot and root at 0 and 12 dS/m. Dried leaves and roots from each biological replicate were pooled, ground and analyzed by a flame photometer 410 (Sherwood, UK) after 48 h of extraction with 1N HCl following the procedure described by Yoshida et al. (1976). Concentrations were expressed as percent of dry weight. The potassium to sodium ratio was also determined. For the reproductive stage, the grains of transgenic and WT plants were sun-dried for 1 week and then dried in an oven at 70°C for 72 h. K^+^ and Na^+^ content of the flag leaves (leaf 0), shoot (all leaves except leaf 3 and the stalk) and third leaf (leaf 3) of pooled biological replicates were separately measured as above. The existence of significant effects due to the transgene *OsNHX1* was determined by the *t*-test.

### Data Analysis

All statistical analyses were done using data analysis ToolPak of Microsoft Office Excel 2007. The F test was performed to verify equal variance of the independent set of samples and based on that result Student’s *t*-test and ANOVA was performed, assuming equal variance or unequal variance as applicable, to compare significant differences (*P* > 0.05) between the transgenic and the WT lines.

## Results

### *Agrobacterium* Mediated Transformation and Confirmation of Transgene Integration

**FIGURE 1:**
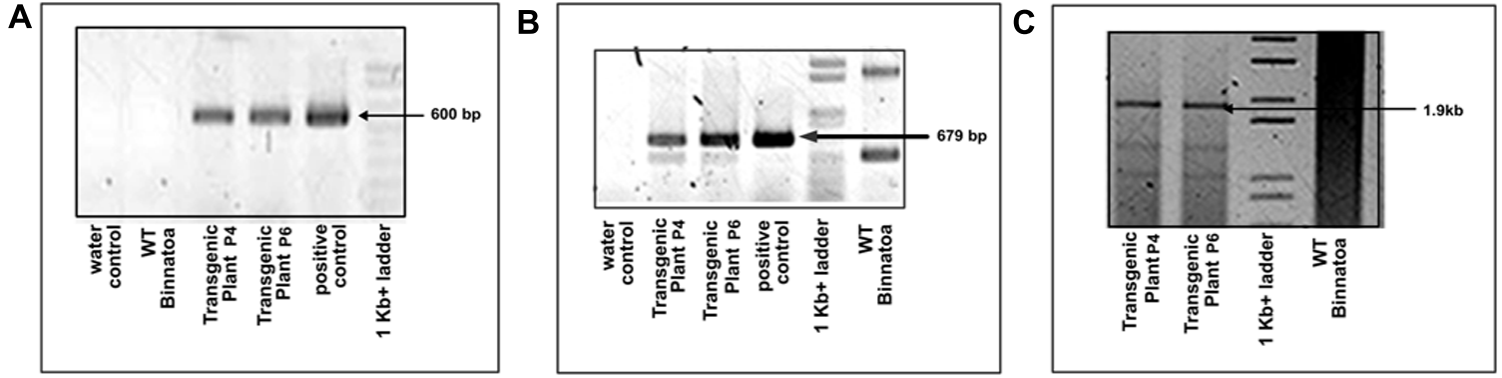
**Confirmation of transgene (*OsNHX1* (1.9 kb)) integration by PCR amplification in the T_1_ plants with **(A)***hpt* gene specific primers, **(B)***OsNHX1* gene specific internal primers, and **(C)** full length *OsNHX1 (1.9 kb)* gene specific primers**.

**FIGURE 2:**
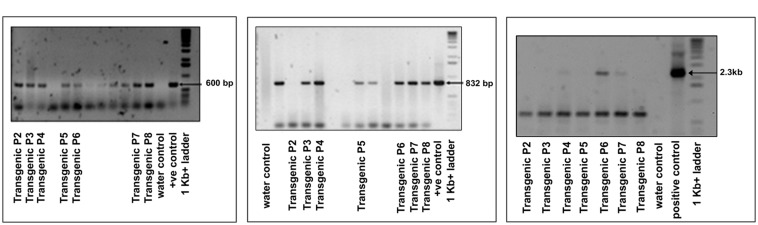
**Confirmation of transgene (*OsNHX1* (2.3 kb)) integration by PCR amplification in the T_1_ plants with **(A)***hpt* gene specific primers, **(B)***OsNHX1* gene specific internal primers, and **(C)** full length *OsNHX1(2.3 kb)* gene specific primers**.

**FIGURE 3:**
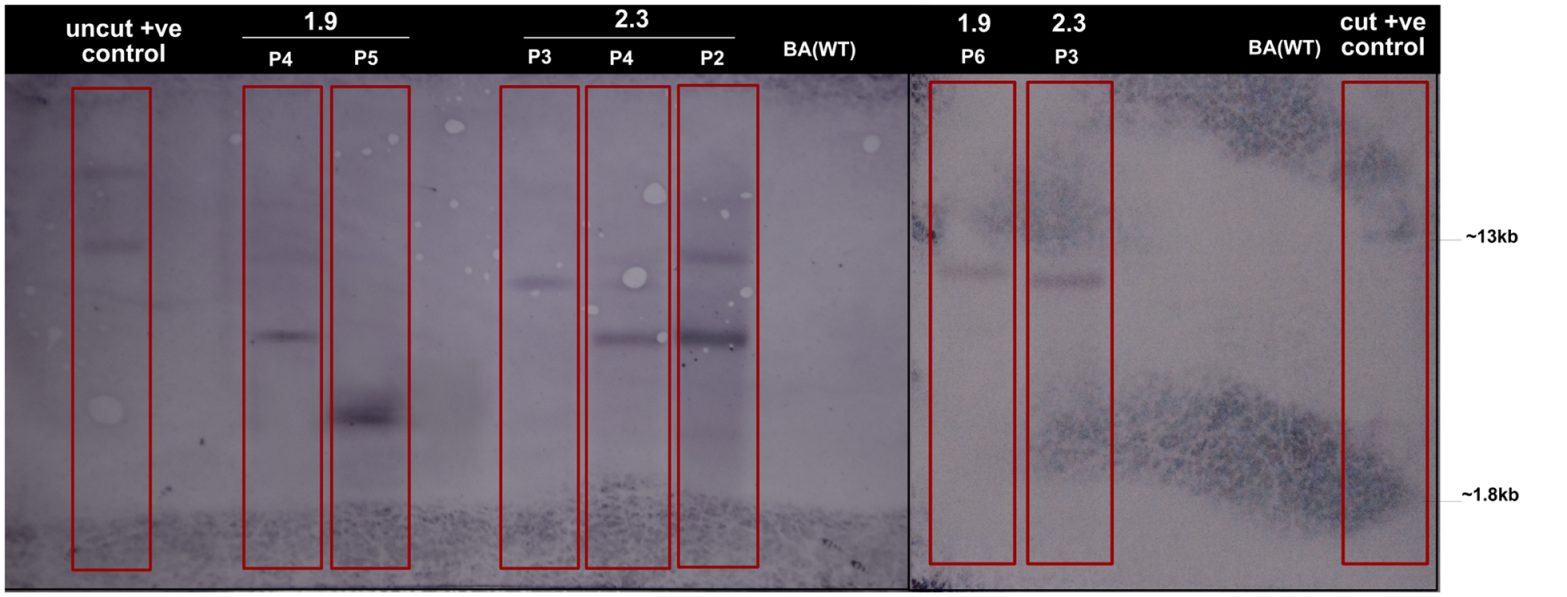
**Confirmation of the transgene from both transformation events in the selected T_3_ plants by Southern Hybridization.** Genomic DNA (20 μg) from selected lines generated from both transformation events (P4, P5, and P6 lines from 1.9 plants and P2, P3, and P4 lines from 2.3 plants) were digested with *BamH1* for DNA blot hybridization. From 1.9, all lines showed single copy and from 2.3, P3, and P4 lines showed single and P2 line showed double copy insertion of transgene whereas no bands were found in the wild type (WT) plants. The positive control, cut singly with *Bam*H1, shows a band of 13 kb, which is the size of the vector.

Tissue culture responsive Binnatoa was transformed with both the constructs separately. The first transformation was conducted with *CaMV_OsNHX1* (1.9 kb) and the second transformation with *CaMV_OsNHX1* (2.3 kb). Positive transformants from both events were selected and regenerated (**Supplementary Material 2**). Only the 1.9 kb cDNA transformation event is shown). Six positively confirmed lines from *CaMV_OsNHX1* (1.9 kb) and seven lines from *CaMV_OsNHX1* (2.3 kb) were generated at the T_0_ stage and advanced to T_1_ and T_2_ generations through *hygromycin* selection and PCR analysis (data not shown). The integration of *OsNHX1_1.9* and *OsNHX1_2.3* in transformed plants was confirmed by PCR with selection marker (*hpt*) specific primers, internal and the full length *OsNHX1* primers (1.9 and 2.3 kb, respectively) (**Figures 1** and **2**). Finally, successful integration of transgene into whole genome was confirmed by Southern hybridization at T_3_ for both the transgenic lines (**Figure 3**). Only single copy insertion transgenic lines (P4 and P6 lines from 1.9 and P3 and P4 lines from 2.3) were selected for further analysis.

### Expression Efficiency of the Transgene and Selection of Best Transformed Plants

**FIGURE 4:**
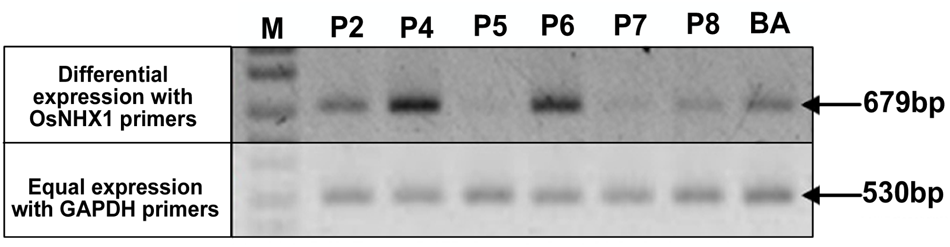
**Expression analysis of *OsNHX1* transgene in WT BA and *CaMV-OsNHX1* (1.9) lines.** Expression intensity of the *OsNHX1* transgene was compared with a house keeping gene, *GAPDH* (GlycerAldehyde-3-Phosphate Dehydrogenase). Transgenic plants showed differential expression compared to the *GAPDH* expression. Only two transgenic lines (P4, P6) showed increased expression compared to WT.

**FIGURE 5:**
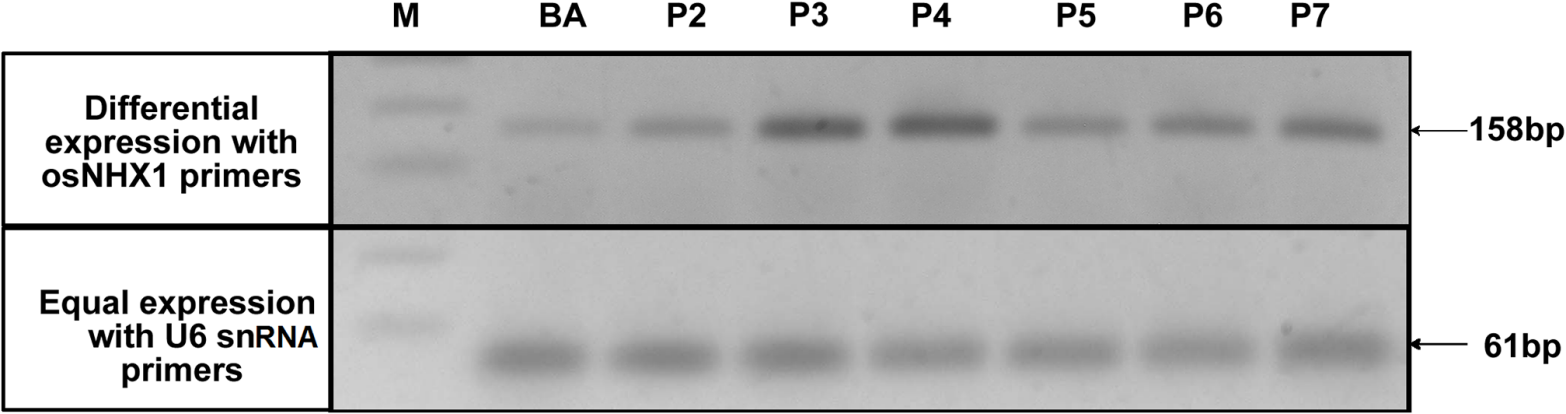
**Expression analysis of *OsNHX1* transgene in WT BA and *CaMV-OsNHX1* (2.3) lines.** Reference gene (U6 snRNA) was used as a control in the semi-quantitative reverse transcriptase PCR. Here, two transgenic lines (P3, P4) showed higher expression of *OsNHX1* compared to other lines and WT BA.

**FIGURE 6:**
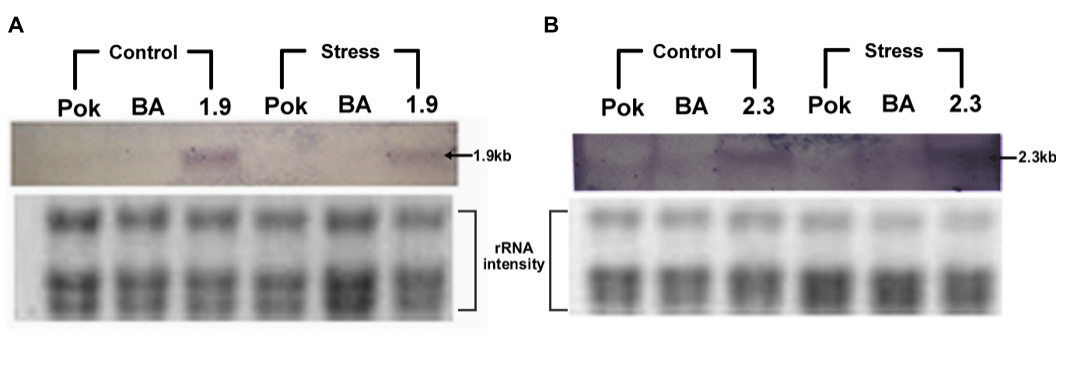
**Northern hybridization of control and stressed samples of salt tolerant Pokkali (Pok), Binnatoa (BA), transgenic 1.9 **(A)**, and transgenic 2.3 **(B)** with Transcript 2-specific *OsNHX1* probe.** Only the 2.3 kb transgenic line showed higher expression of *OsNHX1* gene under salt stress.

In each generation of the transgenic lines, transgene expression was observed using semi-quantitative reverse transcriptase PCR which allowed the confirmation of overexpression of the transgene compared to the WT as well as selection of plants for generation advancement. PCR bands with high intensities were observed after 25 cycles of PCR after the first RT-step in the transgenic plants BA_1.9 (**Figure 4**) and BA_2.3 (**Figure 5**). The bands observed for the WT Binnatoa were very faint compared to the transgenic plants. Enhanced expression of *OsNHX1* in *CaMV-OsNHX1*-1.9 plants, were found in P4 and P6 In the case of *CaMV-OsNHX1*-2.3, P3 and P4 showed enhanced expression compared to WT BA. Northern hybridization with the *OsNHX1* specific probe clearly confirmed the overexpression of the gene in both transgenic BA_1.9 and BA_2.3 under control conditions (**Figure 6**). However, increased expression of the *OsNHX1* gene under 100 mM salt stress (NaCl) was observed only in case of the 2.3 kb transgenic line (**Figure 6B**).

### Seedling Stage Phenotypic Screening Under Salt Stress

Salinity screening at seedling stage was performed at T_2_ for both *CaMV-OsNHX1*-1.9 and *CaMV-OsNHX1*-2.3 transgenic lines to select the best performing plants. Leaf damage score was calculated for both the transgenic events. Transgenic lines P4 and P6 from *CaMV_OsNHX1*-1.9 and P3 and P4 from *CaMV_OsNHX1*-2.3 showed the lowest LDS score and these plants were advanced to the T_3_ stage and subjected to additional screening (**Supplementary Material 3**).

### Leaf Damage Score (LDS)

**FIGURE 7:**
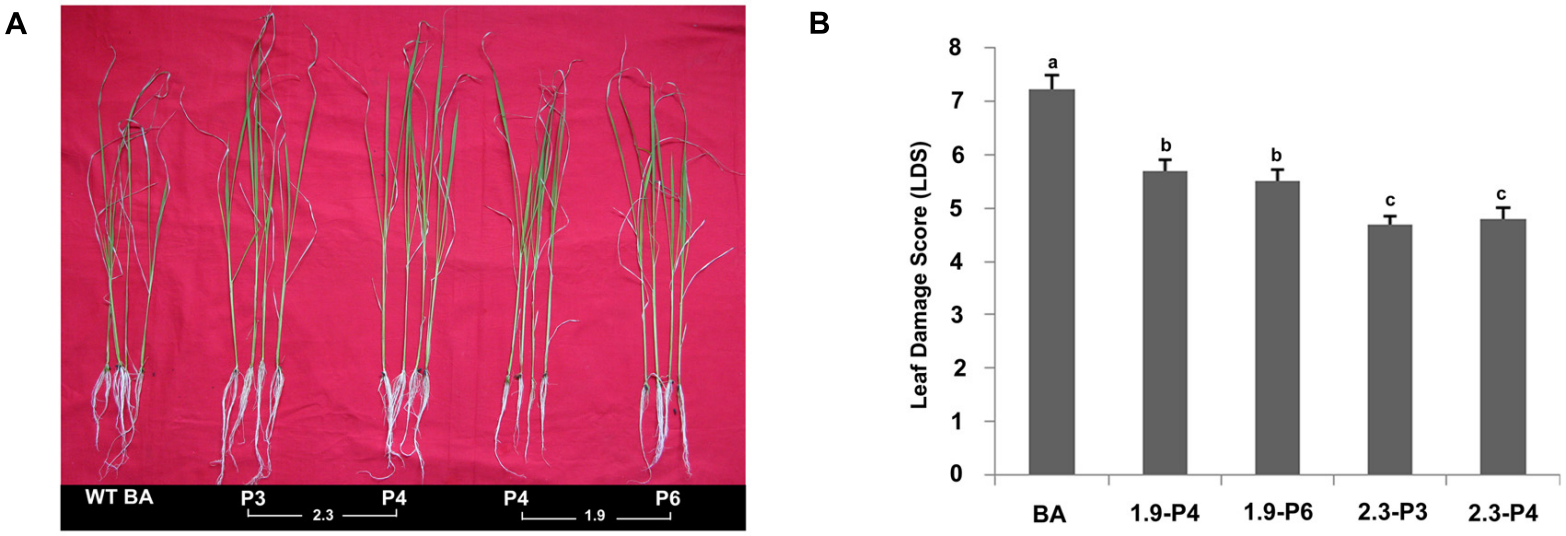
**Physiological screening at the T_3_ stage.** **(A)** Transgenic plants showing better growth under salt stress. BA is the WT plant, 2.3 denotes the transgenic plant transformed with *CaMV-OsNHX1*-2.3 construct, 1.9 denotes transgenic plant transformed with *CaMV-OsNHX1*-1.9 construct. **(B)** Leaf damage score (LDS) at the T_3_ stage. LDS showed significant reduction in both types of transgenic plants compared to WT BA. The 2.3 plants performed better compared to both 1.9 and WT BA. Each bar represents the mean ± SE (*n* = 5). Different letters in each graph (a–c) indicate significant differences (*P* < 0.05, ANOVA and Duncan test).

The selected progenies of from the 2.3 (P3 and P4) and 1.9 kb (P4 and P6) transgenic plants were subjected to physiological screening at the T_3_ stage under salinity stress at 120 mM for 10 days. Both *CaMV-OsNHX1* (1.9 kb) and *CaMV-OsNHX1* (2.3 kb) plants showed better growth morphology than the WT Binnatoa. However, *CaMV_OsNHX1* (2.3 kb) plants showed better performance than the *CaMV_OsNHX1* (1.9 kb) (**Figure 7A**). Leaf damage scores under salt stress were observed for the WT and transgenic plants. The transgenic lines (*OsNHX1*_1.9 and *OsNHX*1_2.3) showed significant reduction in LDS compared to the WT plants (**Figure 7B**). But lowest LDS was observed in the transgenic plants containing *CaMV_OsNHX1*-2.3.

### Electrolyte Leakage and Estimation of Chlorophyll

**FIGURE 8:**
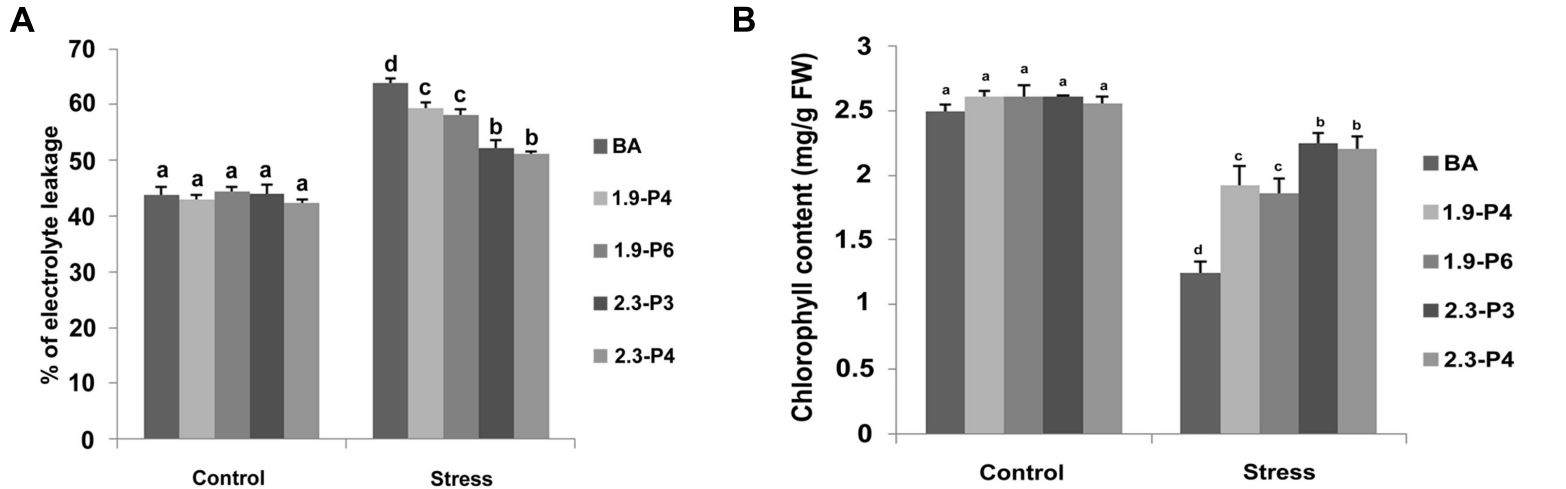
**Electrolyte leakage assay **(A)** and Chlorophyll content measurement assay **(B)** under control and stress (NaCl) condition.** Transgenic plants showed significantly better performance compared to the WT under salt stress. However, the transgenic lines generated from *CaMV_OsNHX1*-2.3 showed better performance compared to *CaMV_OsNHX1*-1.9 transgenic lines. Each bar represents the mean ± SE (*n* = 5). Different letters in each graph (a–c) indicate significant differences (*P* < 0.05, ANOVA and Duncan test).

The percent of electrolyte leakage in the transgenic plants was significantly lower compared to WT. Lower amount of electrolytes leaked in both the *CaMV_OsNHX1* (2.3 kb) plants and *CaMV_OsNHX1* (1.9 kb) plants compared to the WT (**Figure 8A**) under salt stress. In case of the *CaMV_OsNHX1* (2.3 kb) transgenic lines, the electrolyte leakage under 120 mM salinity stress at seedling stage was close to its level without stress (**Figure 8A**).

Estimation of chlorophyll (which was taken as an index of the damage done to the photosynthesis apparatus under stress) indicated that under high salinity conditions, the transgenic lines could retain significantly higher amount of chlorophyll compared to their respective WT (**Figure 8B**), thus documenting the importance of *OsNHX1* over-expressing lines over the non-transgenic lines in maintaining a better metabolic status. Moreover, chlorophyll content in the *CaMV_OsNHX1* (2.3 kb) transgenic lines was significantly higher than that in *CaMV_OsNHX1* (1.9 kb) (**Figure 8B**).

### Reproductive Stage Screening

#### Yield Data

The best selected line from each 1.9 and 2.3 kb transgenic plants (P4 for 1.9 and P4 for 2.3) were subjected to reproductive screening at T_3_ with WT Binnatoa as control. The data were collected under control and salt stress (NaCl) condition. Agronomic traits like tillers per plant, panicle number, panicle height, filled grain per panicle, number of grains per panicle and 1000 grain weight were similar in WT BA and both transgenic plants under control conditions without stress (**Table 1**). However both the transgenic lines showed significantly better performance compared to WT BA in all 6 agronomic traits under continuous 6 dS/m NaCl stress in soil.

**Table 1:** Yield data of wild type (WT) BA and transgenic lines (1.9 and 2.3) under 6 dS/m NaCl stress in soil.

	Name of plants	No. of tillering per plant	Panicle Number per plant	Panicle Length (cm)	No. of Grain per panicle	Filled grain per panicle	1000 grain weight (g)
Control	BA	18.66 ± 1.20	19.66 ± 2.90	14.96 ± 0.79	52.25 ± 6.35	32.98 ± 3.64	19.60 ± 0.24
	1.9	14 ± 1.15	19.66 ± 2.40	13.66 ± 0.78	47.35 ± 3.06	42.19 ± 8.32	19.05 ± 0.34
	2.3	16.66 ± 1.76	17.33 ± 0.88	15.76 ± 0.67	50.06 ± 4.78	38.82 ± 5.05	19.27 ± 0.26
Stress	BA	10 ± 0.40	6 ± 0.40	15.27 ± 0.60	34.94 ± 1.61	13.03 ± 1.57	12.61 ± 0.31
	1.9	12 ± 0.64	9 ± 0.47ˆa	14.75 ± 0.24	41.18 ± 1.65	18.71 ± 0.29 ^a^	14.13 ± 0.21^a^
	2.3	13 ± 1.08ˆb, 1	12 ± 0.47ˆc, 2	16.42 ± 0.85	52.5 ± 2.22	25.71 ± 1.09 ^c,2^	16.66 ± 0.93^b,1^


*Values are mean ± SE (*n* = 5 for stress and *n* = 3 for the control) ^a,b,c^ denotes significant difference between WT and transgenic variety under stress at *P* < 0.05, *P* < 0.01, *P* < 0.001.*

*^1,^^2^ denotes significant difference between 1.9 and 2.3 at *P* < 0.05, *P* < 0.01.*

**FIGURE 9:**
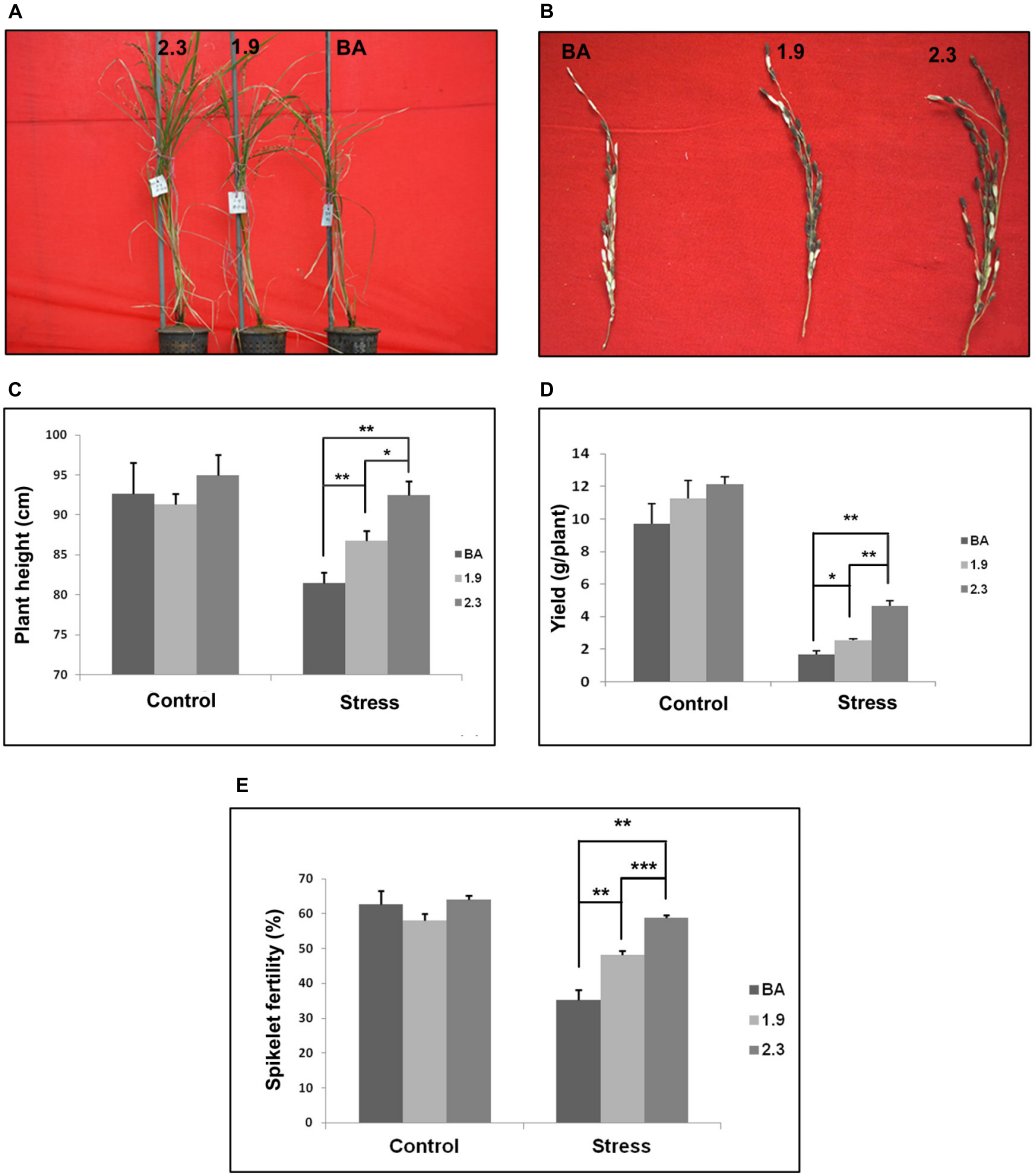
**Measure of Agronomic traits of both transgenic lines (1.9 and 2.3) with corresponding WT BA under continuous salinity (NaCl) stress at 6 dS/m.** **(A)** Phenotype of transgenic lines (1.9 and 2.3) and corresponding WT BA under stress condition at maturity. **(B)** The panicles of transgenic lines and WT BA rice plants collected after salinity stress. Plant Height **(C)**, Yield **(D)**, and Spikelet Fertility **(E)** of transgenic lines and WT BA rice plants under control and salinity stress. In every case, transgenic lines generated from *CaMV_OsNHX1*-2.3 performed significantly better than the *CaMV_OsNHX1*-1.9 transgenic line. ^∗^Significant difference between wild type and transgenic variety and between the two transgenics at *P* < 0.05. ^∗∗^Significant difference between wild type and transgenic variety and between the two transgenics at *P* < 0.01. ^∗∗∗^Significant difference between wild type and transgenic variety and between the two transgenics at *P* < 0.001.

Moreover, the line generated by integrating *CaMV_OsNHX1* (2.3 kb) showed significantly improved tolerance in most of the agronomic traits under continuous salt stress (**Table 1**). The plant height and spikelet number in panicles from *CaMV_OsNHX1* (2.3 kb) was also significantly higher compared to *CaMV_OsNHX1* (1.9 kb) lines under stress (**Figures 9A–C**). Transgenic lines generated from *CaMV_OsNHX1* (2.3 kb) also had significantly improved spikelet fertility rate (58.76%) and higher yield (∼3g/plant) compared to the transgenic lines generated from *CaMV_OsNHX1* (1.9 kb) lines (**Figures 9D,E**).

### Na^+^/K^+^ Ratio at the Seedling and Reproductive Stages

**FIGURE 10:**
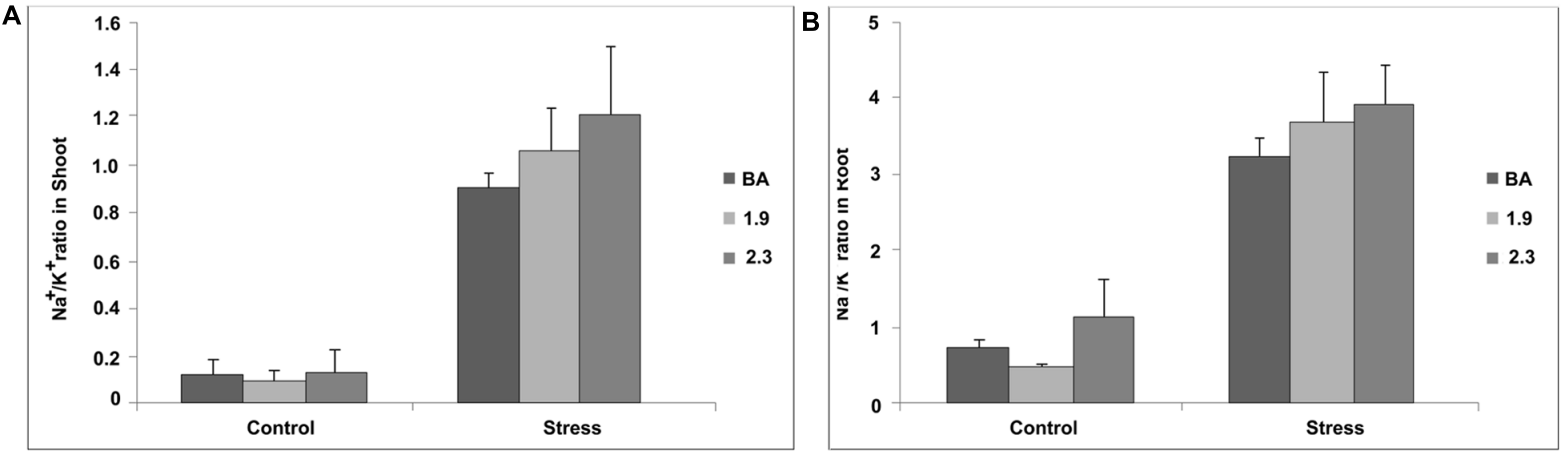
**Na^+^/K^+^ ratio in shoot **(A)** and root **(B)** of wild type BA and transgenic rice seedlings (both *CaMV_OsNHX1*-1.9 and *CaMV_OsNHX1*-2.3) under normal and stress (NaCl) condition at 12 dS/m in hydroponics.** Each bar represents the mean ± SE (*n* = 5).

Transgenic lines and their WT Binnatoa were subjected to Na^+^ and K^+^ content measurement during vegetative and reproductive stage under control and salt (NaCl) stress. At the vegetative stage, *OsNHX1* (2.3 kb) plants maintained the highest Na^+^/K^+^ ratio compared to WT BA and *OsNHX1* (1.9 kb) under stress (**Figure 10**). The overall Na^+^ content increased under stress in both WT and transgenic lines in both shoot and root. However there was no significant relative difference in shoot Na^+^ content between WT BA and its transgenic counterparts when compared to the control values (**Supplementary Material 4**). Interestingly the K^+^ content in the 2.3 transgenic lines doubled under stress compared to a slight increase in the case of the 1.9 transgenic line and lack of change in the WT.

**FIGURE 11:**
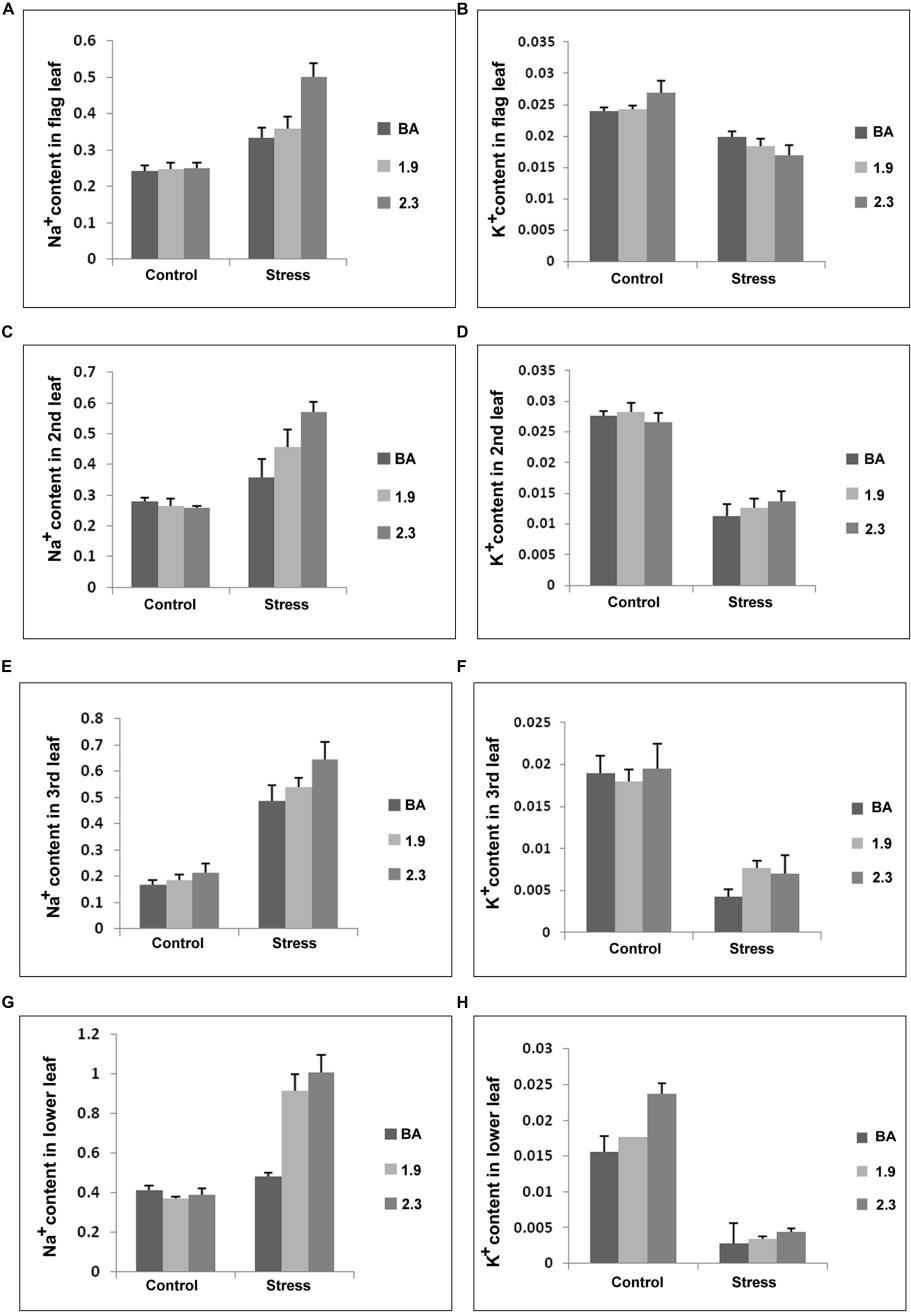
**Na^+^ and K^+^ content (mmol/g DW) **(A–H)** in different leaves of wild type BA and transgenic plants, overexpressing *OsNHX1* (1.9 and 2.3) under continuous salinity (NaCl) stress of 6 dS/m.** Each bar represents the mean ± SE (*n* = 6). Na^+^ and K^+^ were extracted from the different leaves, and ion contents were determined as shown in Section “Materials and Methods”.

The Na^+^ and K^+^ concentrations were also measured in four morphologically different leaves at maturity in WT BA and transgenic lines (BA_1.9 and BA_2.3) under control and continuous stress (NaCl) at 6 dS/m. Under stress, Na^+^ content in the different leaves from both transgenic lines were higher than in WT BA. Moreover, the *CaMV_NHX1* (2.3) lines had higher Na^+^ content in the different leaves compared to the *CaMV_NHX1* (1.9) lines. Notably both the transgenics had twice the Na^+^ content in the lower leaves compared to WT under stress, with the 2.3 line having the highest content. Interestingly, the K^+^ content in the flag leaves for both WT and transgenics was not lowered significantly under stress and maintained preferentially in the upper leaves. Moreover the K^+^ content in the second, third, and lower leaves were higher in both the transgenics compared to WT BA (**Figure 11**). As expected, both WT BA and transgenic lines showed similar concentration of Na^+^ and K^+^ in the tested leaves at reproductive stage without stress.

## Discussion

Seedling and reproductive stages are the two stages at which the rice plant is most susceptible to salinity stress (Moradi and Ismail, 2007). Transformation with the rice vacuolar antiporter gene has mostly been reported to provide tolerance at the seedling stage in rice. The coding sequence (CDS) of the rice vacuolar antiporter gene (*OsNHX1*) has been overexpressed in rice (Fukuda et al., 2004; Chen et al., 2007) imparting salt tolerance only at the seedling level. It has also been reported to give high tolerance to perennial ryegrass but again at the seedling stage only (Wu et al., 2005). Coding sequences from *Atriplex gmelini* and *S. salsa* have also been shown to provide a high level of tolerance to rice only at the seedling stage (Ohta et al., 2002; Zhao et al., 2006). The first report of reproductive stage salt tolerance in rice was reported with the coding sequence of the *P. glaucum* Pg*NHX1* (Verma et al., 2007) but not *OsNHX1*. They, however, only reported setting of seeds at 150 mM NaCl and did not provide a measure of the percent of yield loss. On the other hand, reproductive stage tolerance in rice with a third of the yield using the *OsNHX1* gene from *Nipponbare* was shown under 60 mM NaCl stress (Biswas et al., 2015). The grain yield was found to be twice that of the WT under the same stress. In the latter work, the CDS as well as the 5′ UTR (1.9 kb) of the *OsNHX1* gene was used to transform the rice.

In the current work, we show that when both the 5′ and the 3′ UTR are transformed along with the CDS of the *OsNHX1*gene (2.3 kb), a significant further enhancement is achieved in the level of reproductive salt tolerance compared to the 1.9 kb event. Here the *OsNHX1* cDNA along with either the 5′ UTR or both the 5′ and the 3′ UTR from the rice landrace Pokkali was used for transformation. Interestingly, we found that the level of tolerance from 1.9 kb events originating from *Nipponbare* and *Pokkali* both of which contained only the 5′ UTR, was similar at both the seedling and reproductive stages (Biswas et al., 2015). At the seedling stage, it was found that only the chlorophyll retention was significantly more (*P* < 0.05) in the 2.3 kb event compared to that of the 1.9 kb (**Figure 8**). At the reproductive stage, however, the spikelet fertility (*P* < 0.001) and yield per plant (*P* < 0.01) was significantly more in the complete cDNA transformation event (**Figure 9** and **Table 1**). This was also reflected in the number of panicles per plant and number of filled grains per panicle in the 2.3 kb plants compared to the 1.9 kb ones (**Table 1**). Interestingly, northern blot analysis showed that the expression of the transgene in the 2.3 kb event doubled under stress whereas its expression in the 1.9 kb one remained the same under control and stressed conditions (**Figure 6**).

There was a striking increase in Na^+^/K^+^ ratios after 120 mM stress at the seedling stage in the WT and both the 1.9 and 2.3 kb transgenics (**Figure 10**). The K^+^ levels in the WT, 1.9 and 2.3 kb transgenics under control conditions was observed to be progressively lower, respectively. When stress was applied, however, the K^+^ level remain unchanged in the WT, increased slightly in the 1.9 kb event and nearly tripled in the 2.3 kb event (**Supplementary Material 4**). Therefore it seems that the transgene is somehow helping in the retention of K^+^ in the shoots. The latter seems consistent with the role of the vacuolar *NHX1* antiporter gene in K^+^-mediated osmoregulation, cell growth and plant development (Bassil et al., 2011; Barragán et al., 2012) even though the mechanism remains unclear.

At reproductive stage stress, Na^+^ was shown to be selectively retained in the lower leaves in both the transgenics, where its concentration doubled under stress compared to WT. On the other hand there was no change in the Na^+^ content of the lower leaves between control and stress conditions in the WT (**Figure 11**). This could be explained by the higher activity of the transgenes due to the larger vacuoles present in the older lower leaves (Wang et al., 2012). There was no difference in the relative Na^+^ contents in the 2^nd^ and 3^rd^ leaves between WT and the transgenics. However the Na^+^ content in the flag leaf was the highest in the 2.3 kb transgenic. Interestingly the K^+^ content was found to be progressively reduced starting from the flag, second, third to the lower leaves in both WT and the transgenics. Or in other words K^+^ retention occurred in the flag leaves at the expense of the others. However, this was true for both the WT and transgenics and such partitioning to retain the beneficial K^+^ in photosynthetically active tissues may be a property of the slightly saline tolerant WT rice landrace, Binnatoa.

The *OsNHX1* gene has three transcripts as explained in the introduction (LOC_Os07g47100, gramene). During our cloning procedure for the *OsNHX1*gene the forward primer that we had used during cDNA synthesis corresponded to the reported upstream region by Fukuda et al. (1999) (NCBI Accession AB021878). This had pulled out transcript 2 which has an extended 5′ UTR. However, the size of the reported upstream and downstream untranslated regions in this Accession, were shorter than that found in the latest gramene release. Therefore we ended up cloning 2311 bp rather than 2394 bp. When a 679 bp probe common to all three transcripts was used to probe northerns between the WT, the two transgenics, as well as *Nipponbare* and *Pokkali*, only the transgenics were observed to show multiple transcripts ranging from 1.6 to 2.4 kb in both control and stressed conditions. Interestingly the pattern of transcripts was different between the 1.9 and 2.3 kb transgenics. These bands could either be the alternative transcripts of *OsNHX1* or different members of the OsNHX1-OsNHX5 family (Fukuda et al., 2011), **Supplementary Material 5** and **Tables 5.1** and **5.2**). In a separate semi-quantitative PCR, transcript 3 was found to be upregulated in both the 1.9 and 2.3 kb transgenics. Therefore, it seems that over expression of a truncated transcript 2 (1.9 kb) and the complete one 2.3 kb somehow is regulating the expression of either the alternative transcripts or their homologs. How this is bringing about greater tolerance in the 2.3 kb transgenic is not clear. However as mentioned earlier the expression in the 2.3 kb plant under salt stress increases significantly compared to the pattern of no-change under control and stressed conditions in the 1.9 kb plant (**Figure 6**).

Our work therefore shows that, during transformation, one should use the complete transcripts instead of only the coding region. This may be very important in increasing the efficiency of the transgene. This may be particularly important for genes involved in stress tolerance, many of which are alternatively spliced (Ding et al., 2014).

## Author Contributions

UA and SB performed molecular confirmation, did some comparative physiology, compiled the data, and did statistical analyses. UA and SB have equal contribution as author. SR and ZS wrote the manuscript. SE and RM compiled molecular and physiological data from different generations, SE and TH did expression analysis in transgenic lines. ZS designed the study.

## Conflict of Interest Statement

The authors declare that the research was conducted in the absence of any commercial or financial relationships that could be construed as a potential conflict of interest.
