# Supplementary material for: Enhanced Salt Tolerance Conferred by the Complete 2.3 kb cDNA of the Rice Vacuolar Na+/H+ Antiporter Gene Compared to 1.9 kb Coding Region with 5′ UTR in Transgenic Lines of Rice
Source: Front Plant Sci. 2016 Jan 25;7:14. doi: 10.3389/fpls.2016.00014 (PMC4724728; doi:10.3389/fpls.2016.00014)
Supplement: Supplementary file 1 [file Data_Sheet_1.DOCX]

**Supplementary Material 1.1:** Primer sequences used in the study.

| **Primer Name** | **Sequence** | **Product Size** |
| --- | --- | --- |
| 2.3F | 5 ‘ CACCGAAGAGAGTTTTGTAGCGAG 3’ | 2311bp |
| 2.3 R | 5 ‘ CAACCAAGCAAAATTATTATAC 3’ |  |
| OsNHX1_1900F | 5 ‘ GAGAAGAGAGTTTTGTAGCGA 3’ | 1904 bp |
| OsNHX1_1900R | 5 ‘ TCATCTTCCTCCGTGACTCTGC 3’ |  |
| HPT -F | 5 ‘ GATGTTGGCGACCTCGTATT 3’ | 809 bp |
| HPT -R | 5 ‘ GCGAAGAATCTCGTGCTTTC 3’ |  |
| OsNHX1-679F | 5 ‘ GCTGGATTGCTCAGTGCATA 3’ | 809 bp |
| OsNHX1-679R | 5 ‘ AAGGCTCAGAGGTGACAGGA 3’ |  |
| OsNHX1_832_F | 5‘ GGCATTCACCAACGAGCAAG 3 ‘ | 832bp |
| OsNHX1_832_R | 5‘ TATGTATGCACTGAGCAAT 3 ‘ |  |
| Trans_2_northern_F | 5 ‘ GAAGCCAACCGAGAGAGGTC 3’ | 158bp |
| Trans_2_northern_R | 5 ‘ GAATTAGGCATTCACCAACGA 3’ |  |
| G3PDH_F | 5 ‘ GCAGGAACCCTGAGGA 3 ‘ | 530bp |
| G3PDH_R | 5‘ TTCCCCCTCCAGTCCT 3 ‘ |  |
| U6_snRNA_F | 5‘ TACAGATAAGATTAGCATGGCCCC 3 ‘ | 61bp |
| U6_snRNA_R | 5‘ GGACCATTTCTCGATTTGTACGTG 3‘ |  |

**NHX1 Transcript 2 specific probe sequence: 158 bp**

GAAGCCAACCGAGAGAGGTCTCGATACCAAATCCCGATTTCTCAACCTGAATCCCCCCCCCACGTTCCTCGTTTCAATCTGTTCGTCTGCGAATCGAATTCTTTGTTTTTTTTTCTCTAATTTTACCGGGAATTGTCGAATTAGGCATTCACCAACGA

**Supplementary Material 1.2:**


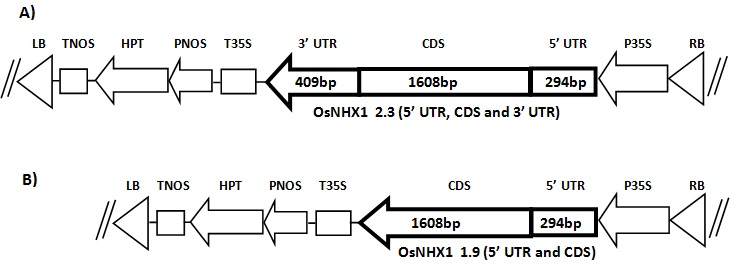


**Figure:** Schematic diagram of T-DNA region of pH7WG2_OsNHX1 2.3 (A) and pH7WG2_OsNHX1 1.9 expression vector, RB right border; P35S cauliflower mosaic virus 35S promoter; 5’UTR: 5’ untranslated region; CDS: coding sequence; 3’UTR: 3’ untranslated region; T35S: cauliflower mosaic virus 35S terminator; PNOS: NOS promoter; HPTII: Hygromycin resistant gene; TNOS: NOS terminator; LB: Left border
