# Supplementary material for: Enhanced Salt Tolerance Conferred by the Complete 2.3 kb cDNA of the Rice Vacuolar Na+/H+ Antiporter Gene Compared to 1.9 kb Coding Region with 5′ UTR in Transgenic Lines of Rice
Source: Front Plant Sci. 2016 Jan 25;7:14. doi: 10.3389/fpls.2016.00014 (PMC4724728; doi:10.3389/fpls.2016.00014)
Supplement: Supplementary file 2 [file Data_Sheet_2.DOCX]

**Supplementary Material 2 :**


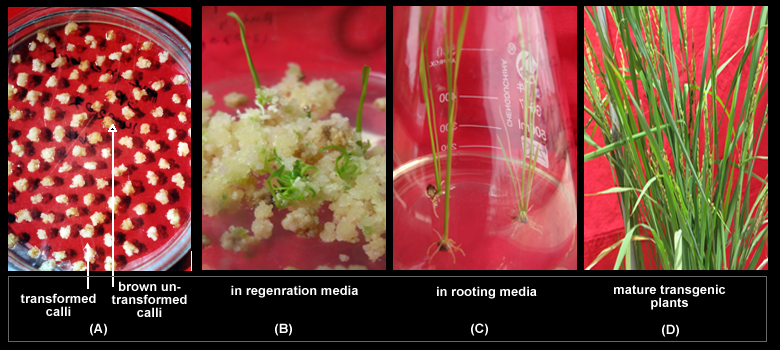


Transformation steps for the *CaMV_OsNHX1*-1.9 construct**:** Calli in selection media a few days after infection and selection (A), Plantlets in regeneration media (B), growth of transformed plants in rooting media (C) and the mature transgenic plants (D).
