# Supplementary material for: Enhanced Salt Tolerance Conferred by the Complete 2.3 kb cDNA of the Rice Vacuolar Na+/H+ Antiporter Gene Compared to 1.9 kb Coding Region with 5′ UTR in Transgenic Lines of Rice
Source: Front Plant Sci. 2016 Jan 25;7:14. doi: 10.3389/fpls.2016.00014 (PMC4724728; doi:10.3389/fpls.2016.00014)
Supplement: Supplementary file 3 [file Data_Sheet_3.DOCX]

**Supplementary Material 3:**

**Phenotypic screening of the CaMV-OsNHX1-2.3 transgenic plants at T_1_ and T_2_ stage**

T_2_ Seeds from *CaMV-OsNHX1*-1.9 transgenic plants and both T_1_ and T_2_ seeds from *CaMV-OsNHX1*-2.3 were germinated and set for screening with three check varieties (Pokkali and BA). All transgenic lines showed significantly better tolerance in terms of leaf damage scores (Fig supplementary 3a and b) than the wild type BA under salinity stress at 120 mM NaCl for 12 days. It was observed that the two lines (P4 and P6 from 1.9; P3 and P4 from 2.3) from both gene-constructs (1.9 and 2.3) showed better tolerance compared to the other lines.


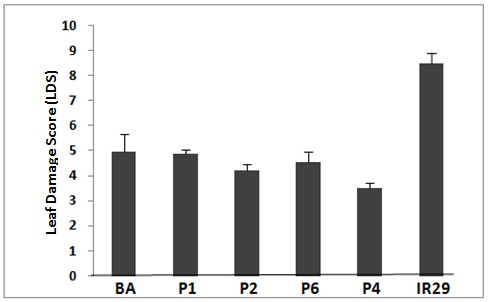


**Figure** supplementary 3. (a) Transgenic rice with *CaMV-OsNHX1*-1.9 at T_2_ and (b) Transgenic rice with *CaMV-OsNHX1*-2.3 at both T_1_ and T_2_ stage performed better at physiological screening compared to wild type BA. SES Score of wild-type and transgenic rice seedlings after NaCl stress at 12 dS/m in hydroponics. Each bar represents the mean ± SE (n = 18)
