# Supplementary material for: Enhanced Salt Tolerance Conferred by the Complete 2.3 kb cDNA of the Rice Vacuolar Na+/H+ Antiporter Gene Compared to 1.9 kb Coding Region with 5′ UTR in Transgenic Lines of Rice
Source: Front Plant Sci. 2016 Jan 25;7:14. doi: 10.3389/fpls.2016.00014 (PMC4724728; doi:10.3389/fpls.2016.00014)
Supplement: Supplementary file 4 [file Data_Sheet_4.DOCX]

**Supplementary Material 4:**

**K^+^ content (mmol/g dry wt) in shoot**

**Na ^+^ content (mmol/g dry wt) in Shoot**

**Na ^+^ content (mmol/g dry wt) in Root**

**K^+^ content (mmol/g dry wt) in Root**

**Supplementary Figure 4:** Na^+^ and K^+^ contents in shoot (4a, 4c) and root (4b, 4d) of wild-type BA and transgenic rice seedlings (both *CaMV_OsNHX1*-1.9 and *CaMV_OsNHX1*-2.3) under normal and stress (NaCl) at 12 dS/m in hydroponics. Each bar represents the mean ± SE (n=5).
