# Supplementary material for: Enhanced Salt Tolerance Conferred by the Complete 2.3 kb cDNA of the Rice Vacuolar Na+/H+ Antiporter Gene Compared to 1.9 kb Coding Region with 5′ UTR in Transgenic Lines of Rice
Source: Front Plant Sci. 2016 Jan 25;7:14. doi: 10.3389/fpls.2016.00014 (PMC4724728; doi:10.3389/fpls.2016.00014)
Supplement: Supplementary file 5 [file Data_Sheet_5.DOCX]

**Supplementary Material 5:**

Northern hybridization with the probe (679 bp) was designed from a common region of all 3 transcripts of *OsNHX1.* The probe showed multiple bands in the blot. Interestingly these multiple bands were absent in the wild type plants and only present in the transgenics (1.9 and 2.3) (Supplementary Fig 5.1). To explain this observation, the probe was BLASTed with transcriptome data from both the japonica and indica subspecies of rice. (Supplementary Table 5.1). BLAST result revealed that this common probe matched with other family members of NHX (OsNHX1-OsNHX4) (Supplementary Table 5.2) and it is possible that the multiple bands in the transgenics are from other family members or from the other alternative transcripts of OsNHX1. These bands are present in both stressed and control and in both transgenic (1.9 and 2.3 of the OsNHX1 gene) samples. Use of rice rRNA markers (Supplementary Table 5.3) indicated that the bands are in the range of ~1.6 to 2.4 kb (Supplementary fig 5.1) and that is the range where the transcript lengths of the NHX family proteins resides. Due to poor annotation in the indica transcriptome the known available transcript lengths in the databases may be misleading. Also due to multiple annotations, the exact length in the japonica transcriptome also varies. A regulatory mechanism may be embedded in the UTRs that can help over-expression of specific transcripts as can be seen in semi-quantitative RT-PCR using OsNHX1 transcript 3-specific primers (Fig supplementary 5.2). Equal amounts of RNA based on RT-PCR with GADPH (not shown) were loaded.


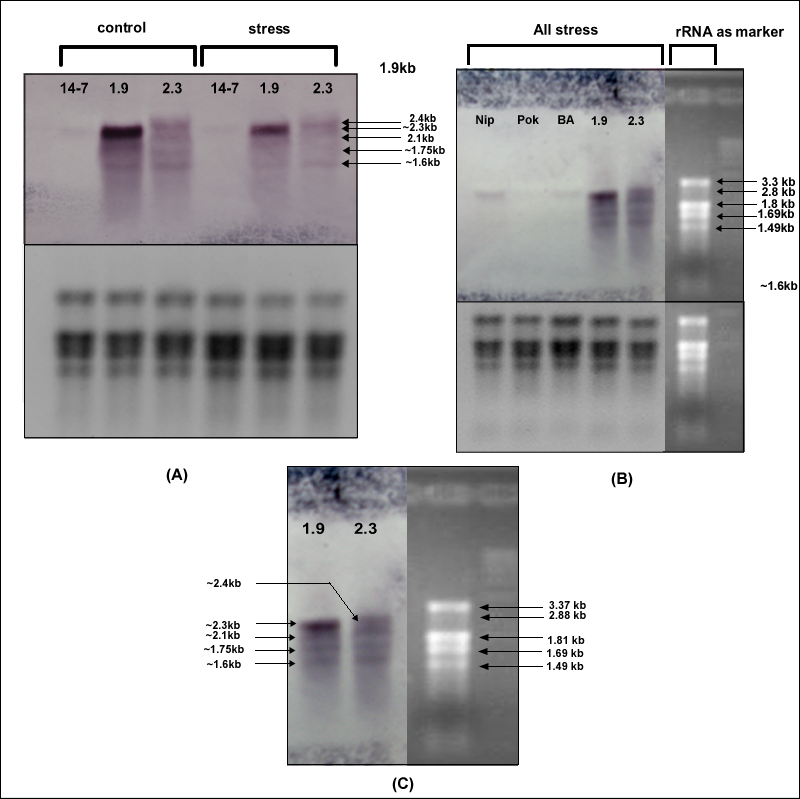


**Fig supplementary 5.1:** Transgene over-expression with UTRs showed multiple bands in the blot

1. Northern blot of different transgenics (BA containing *CaMV-OsNHX1-1.9 and CaMV-OsNHX1-2.3* and 14-7 (BR28 containing CaMV-*OsNHX1-1.9,* Biswas *et. al 2015)* using 679 bp probe under control and 24 hours 100 mM NaCl stress
2. Northern blot of wild type plant (BA, Nipponbare (Nip) and Pokkali (Pok)) and BA containing *CaMV-OsNHX1-1.9 and CaMV-OsNHX1-2.3* transgenics using 679 bp probe under 24 hours 100 mM NaCl stress
3. Blown-up version of part of the gel in B to show the banding pattern obtained in the Northerns of *CaMV-OsNHX1-1.9 and CaMV-OsNHX1-2.3*  transgenic plants


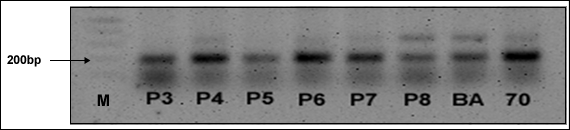


**Fig supplementary 5.2:** Transgenic plants showing greater intensity for OsNHX1 transcript 3 specific product.

*(In P4, P6, P7 and BA-70 the bands at around 200bp are denser. P4, P6 and P7 were selected as positive transgenic plants for the 2.3 transformation. And 70 is another transgenic plants with the 1.9kb transgene (*Biswas *et. al 2015)*

**Table supplementary 5.1:** Blast hits 679bp length probe with japonica and indica transcriptome of rice

| **Match with** | **Chr** | **Score** | **E value** | **%ID** | **Length** | **Transcript size (bp)** | **Description of the match** |
| --- | --- | --- | --- | --- | --- | --- | --- |
| JAPONICA | | | | | | | |
| OS07T0666900-01 | 7 | 3395 | 5.9e-149 | 100 | 679 | 2313 | Sodium/hydrogen exchanger  OsNHX1 transcript2 |
| OS07T0666900-02 | 7 | 3395 | 6.1e-149 | 100 | 679 | 2227 | Sodium/hydrogen exchanger  OsNHX1 transcript1 |
| LOC_Os07g47100.3 | 7 | 1118 | 0 | 89.1 | 605 | 1820 | Sodium/hydrogen exchanger  OsNHX1 transcript3 |
| OS11T0648000-01 | 11 | 1797 | 8.7e-77 | 75.65 | 657 | 2329 | Sodium/hydrogen exchanger  OsNHX2  transcript1 |
| OS11T0648000-02 | 11 | 1797 | 1.1e-76 | 75.65 | 657 | 1887 | Sodium/hydrogen exchanger  OsNHX2 transcript2 |
| OS05T0148600-01 | 5 | 1772 | 1.3e-75 | 75.04 | 661 | 2104 | Sodium/hydrogen exchanger  OsNHX3 |
| OS06T0318500-00 | 6 | 1269 | 8.5e-53 | 67.74 | 651 | 1693 | Sodium/hydrogen exchange  OsNHX4 |
| INDICA | | | | | | | |
| BGIOSGA027380-TA | 8 | 1860 | 1.1e-79 | 76.71 | 657 | 1638 | Sodium/hydrogen exchanger  (match with japonica OsNHX2) |
| BGIOSGA026347-TA | 7 | 1810 | 1.4e-134 | 100 | 362 | 1497 | Putative uncharacterized protein (matches with OsNHX1 ) |
| BGIOSGA026347-TA | 7 | 1315 | 1.4e-134 | 100 | 263 | 1497 | Putative uncharacterized protein (matches with OsNHX1 ) |
| BGIOSGA018801-TA | 5 | 1085 | 3.2e-58 | 72.58 | 445 | 1491 | Putative uncharacterized protein (matches with OsNHX3) |
| BGIOSGA022793-TA | 6 | 791 | 4.8e-31 | 69.95 | 376 | 1512 | Sodium/hydrogen exchanger  (matches with OsNHX4) |
| BGIOSGA018801-TA | 5 | 342 | 3.2e-58 | 73.91 | 138 | 1491 | Putative uncharacterized protein (matches with OsNHX3) |

**Table supplementary 5.2:** Transcript size of other family members of the NHX family

| **Name** | **Other name** | **Genbank**  **Accession** | **MSU ID** | **Chr** | **Transcript Size** |
| --- | --- | --- | --- | --- | --- |
|  |  |  | **IRGSP ID** |  |  |
| OsNHX1 (transcript 1) | OsNHX1-202 | AK064004 | LOC_Os07g47100.1 | 7 | 2265 bp / 2.1kb (Fukuda et al, 2011) |
|  |  |  | OS07T0666900-02 |  |  |
| OsNHX1 (transcript 2) | OsNHX1-201 | AK064004 | LOC_Os07g47100.2 | 7 | 2394 bp /2313 bp * /2227 bp |
|  |  |  | OS07T0666900-01 |  |  |
| OsNHX1 (transcript 3) | Not included in current annotation | - | LOC_Os07g47100.3 | 7 | 1820 bp |
|  |  |  | - |  |  |
| OsNHX2 (transcript 1) | OsNHX2-201 | AK066444 , AB531435 | LOC_Os11g42790.1 | 11 (chr 8 in indica) | 2329 bp / 2.4kb (Fukuda et al, 2011) |
|  |  |  | OS11T0648000-01 |  |  |
| OsNHX2 (transcript 2) | OsNHX2-202 | AK066444 , AB531435 | - | 11 (chr 8 in indica) | 1887 bp |
|  |  |  | OS11T0648000-02 |  |  |
| OsNHX3 | NHX2-201 | AB531433 | LOC_Os05g05590 | 5 | 2108bp / 2104bp /2.3 kb (Fukuda et al, 2011) |
|  |  |  | OS05T0148600-01 |  |  |
| OsNHX4 | P0592B08.3-201 | In clone AP003507 | LOC_Os06g21360 | 6 | 1693 bp |
|  |  |  | OS06T0318500-00 |  |  |
| OsNHX5 | OS09G0286400-201 | AB531434 | OS09T0286400-01 | 9 | 2173 bp / 2163bp / 2.4 kb (Fukuda et al, 2011), |
|  |  |  |  |  |  |

* size of the transgene (2.3) in the current study

**Table supplementary3.3:** rRNA size pattern in *Oryza sativa*

| **rRNA** | **Size in Japonica** | **Reference ncbi id** | **Size in indica** | **Reference ncbi id** | **Encoded by** |
| --- | --- | --- | --- | --- | --- |
| *25s | 3377bp | M11585.1 | - | - | Nuclear |
| *17s | 1812 | X00755.1 | - | - | Nuclear |
| *23s | 2883 | X15901.1 | 2887 | JN861110.1 | Chloroplast |
| *16s | 1490 | X15901.1 | 1490 | JN861110.1 | Chloroplast |
| 26s | 3541 | DQ167400.1 | 3506 | JF281153.1 | Mitochondria |
| *18s | 1694 | DQ167400.1 | 1693 | JF281153.1 | Mitochondria |

* mentioned in Fig 1 as marker
